# Prevention and Treatment of New-Onset Postoperative Atrial Fibrillation in the Acute Care Setting: A Narrative Review

**DOI:** 10.3390/jcm14196835

**Published:** 2025-09-26

**Authors:** Jean-Luc Fellahi, Marc-Olivier Fischer, Martin Ruste, Matthias Jacquet-Lagreze

**Affiliations:** 1 Service d’Anesthésie Réanimation, Hôpital Universitaire Louis Pradel, Hospices Civils de Lyon, 59 Boulevard Pinel, 69500 Lyon, France; martin.ruste@chu-lyon.fr (M.R.); matthias.jacquet-lagreze@chu-lyon.fr (M.J.-L.); 2 Laboratoire CarMeN, Inserm UMR 1060, Université Claude Bernard Lyon 1, 69008 Lyon, France; 3 Institut Aquitain du Cœur, Clinique Saint-Augustin, ELSAN, 33074 Bordeaux, France; marcolivierfischer@yahoo.fr

**Keywords:** new-onset atrial fibrillation, postoperative atrial fibrillation, amiodarone, beta-blockers, landiolol

## Abstract

New-onset postoperative atrial fibrillation (POAF) is common after cardiac and major noncardiac surgery and significantly associated with short- and long-term adverse events. Multiple management strategies have been described but the lack of evidence from large randomized controlled trials and the lack of consensus regarding best practices has led to major variations in practice patterns. Considering on the one hand its serious adverse effects and complex drug interactions, and on the other hand discrepancies among recent international guidelines, the indications of amiodarone to both prevent and treat POAF should be reserved to patients at high risk of POAF only, or patients with hemodynamic instability and/or severely reduced left ventricular ejection fraction. Perioperative optimization of oral and intravenous cardio-selective beta-blockers to prevent POAF, and control heart rate when POAF occurs with a rapid ventricular response is the recommended first-line strategy, simultaneously with the treatment of associated factors. Given their efficient and safe profile, ultra-short-acting intravenous beta-blockers like esmolol or landiolol could be preferentially used in acute care patients. Besides waiting for the results of ongoing RCTs in cardiac and noncardiac surgery, the use of oral anticoagulation in patients with POAF should take into account the individualized thromboembolic/hemorrhagic risk ratio.

## 1. Introduction

Postoperative atrial fibrillation (POAF) is defined as new-onset atrial fibrillation that occurs within the first postoperative days following cardiac and noncardiac surgery. POAF is a common complication that occurs in 30–50% of patients undergoing cardiac surgery [1,2,3], 5–30% of patients undergoing major noncardiac surgery, and 5–45% of critically ill patients [4]. The reported incidence of POAF has remained remarkably steady over recent decades. POAF is a serious complication that increases postoperative cardiac, cerebral, and renal morbidity, short- and long-term mortality, lengths of stay in intensive care unit (ICU) and in hospital, and healthcare costs [5,6,7,8,9]. Numerous risk factors of POAF have been well identified. They can be divided in non-modifiable risk factors related to atrial myopathy and patients’ characteristics and comorbidities (age, gender, obesity, alcohol, sleep apnea, hypertension, valvular disease, and ventricular dysfunction) [10], and in transient modifiable stress factors accompanying acute illness (sympathetic activity, hypo-/hypervolemia, hypo-/hypertension, anemia, pain, electrolyte disturbances, metabolic alterations, hypo-/hyperthermia, hypoxia, and inflammation) [11,12,13]. From a clinical point of view, POAF is a paroxysmal phenomenon whose peak frequency occurs at postoperative day 2 (interquartile range: 1–3) [14]. More than 90% of patients with POAF return to normal sinus rhythm within the first seven postoperative days [5], even if a second acute episode is observed in 20 to 40% of cases [15]. When paroxysmal POAF lasts beyond postoperative day 7, it becomes persistent POAF. Noteworthy, POAF is associated with a 4–5-fold increase in recurrent atrial fibrillation during the next five years [16,17]. POAF is most often hemodynamically well tolerated and rarely responsible for acute circulatory failure or shock state. While multiple strategies to prevent POAF with pre-treatment or acute drug treatment have been described, there is a lack of evidence from large randomized controlled trials (RCTs). Furthermore, various treatment strategies have been described and diversely recommended in numerous international guidelines over the last twenty years. The lack of consensus regarding best practices for the management of POAF has led to major variations in practice patterns [1,18,19,20,21].

## 2. Prevention of POAF

### 2.1. Amiodarone: A Dead Man Walking [22]

**Figure 1:**
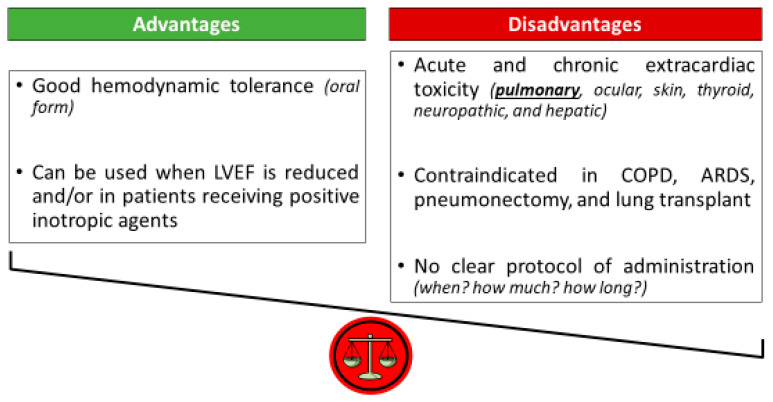
Individual benefit/risk ratio of amiodarone to prevent and/or treat postoperative atrial fibrillation (POAF). ARDS: acute respiratory distress syndrome; COPD: chronic obstructive pulmonary disease; LVEF: left ventricular ejection fraction. Among extracardiac adverse effects, pulmonary toxicity has the most clinically significant impact and can lead to acute respiratory distress syndrome, refractory hypoxemia, and death.

Amiodarone has demonstrated superiority over placebo in preventing POAF after cardiac surgery for a long time [23,24,25,26], and is currently recommended in several international guidelines, especially when beta-blockers are contraindicated [27,28]. The latest 2024 ESC guidelines for the management of atrial fibrillation recommend using perioperative amiodarone where drug therapy is desired to prevent POAF after cardiac surgery (Class I recommendation, level of evidence A) [29]. This is, however, at the cost of potential adverse hemodynamic effects, such as high-grade atrioventricular blocks, refractory hypotension, or cardiogenic shock. In addition, various schemes of administration have been reported, so it is currently impossible to provide a clear and consensual protocol that could be used on a routine basis. When to start amiodarone preoperatively, at which dose, and for how long are unresolved practical questions. Moreover, amiodarone and its metabolites accumulate in tissues in high concentrations and are responsible for both serious adverse non-hemodynamic effects (pulmonary, ophthalmic, hepatic, thyroid, skin, and neuropathic), and numerous drug interactions. In a retrospective study including 137 consecutive patients who underwent lung or heart–lung transplant, the prevalence of POAF within 26 days after surgery was high (45%) and the use of amiodarone was an independent predictor of short-term mortality in multivariate analysis (HR 2.97 [95% CI: 1.19–7.41], *p* = 0.02) [30]. Even though the lungs account for less than 5% of all amiodarone-related complications, pulmonary involvement has the most clinically meaningful impact and can contribute to patients’ mortality [31]. Thus, the individual benefit/risk ratio analysis of amiodarone therapy to prevent POAF pleads most often against its preoperative systematic use, the risks outweighing the benefits (Figure 1) [22]. The joint recommendations for best practice around prevention and management of POAF in patients undergoing cardiac surgery from both Society of Cardiovascular Anesthesiologists and European Association of Cardiothoracic Anaesthetists suggest using prophylactic amiodarone only in patients deemed at high risk of developing POAF (Class IIa recommendation, level of evidence A/B) [32].

### 2.2. Perioperative Optimization of Beta-Blockers

Large cohort studies have previously shown that perioperative optimization of beta-blockers was strongly and independently associated with a reduction in POAF following cardiac and noncardiac surgery, especially in chronically treated patients [5]. Indeed, the withdrawal of treatment was responsible for a two-fold increase in the incidence of POAF, whereas continuing beta-blockers pre- and postoperatively was associated with a marked decrease in POAF (OR 0.49 [95% CI: 0.39–0.61], *p* < 0.001) [5]. In a French multicenter prospective cohort study including 663 cardiac surgical patients, the odds of POAF were significantly reduced by resuming beta-blockers between 72 and 96 h after surgery [33]. Additionally, a Cochrane systematic review of cardiac surgery RCTs reported that patients on beta-blocker therapy had a lower risk of POAF compared with control (RR 0.50 [95% CI: 0.42–0.59]) [34]. Focusing on landiolol, an ultra-short-acting intravenous highly cardio-selective beta-blocker, a systematic review of RCTs found a huge reduction in POAF following cardiac surgery in patients receiving a small dose of landiolol perioperatively when compared with control (13.7% vs. 37.6%, *p* < 0.001) [35]. Interestingly, the hemodynamic tolerance of landiolol was excellent, most often avoiding hypotension and its consequences [35]. Recently, a meta-analysis of RCTs confirmed the beneficial effect of landiolol for the prevention of POAF after cardiac surgery compared to patients allocated to control groups (RR 0.40 [95% CI: 0.30–0.54], *p* < 0.001) [36]. The use of landiolol was also associated with a significant decrease in hospital length of stay. A similar beneficial effect of landiolol was reported in a propensity score-matched analysis including 358 consecutive adult patients scheduled for aortic root, ascending aorta, and aortic arch surgery [37]. In this last study, the incidence of POAF was 18.9% vs. 38.7% (*p* = 0.002) in the landiolol group compared to control. All these studies provide compelling evidence that perioperative optimization of beta-blockers, especially in chronically treated patients, is both efficient and safe in decreasing the incidence of POAF following cardiac and noncardiac surgery, and that perioperative administration of intravenous low-dose landiolol should be considered by physicians as a valuable therapeutic option for POAF prevention.

### 2.3. Other Interventions

In the specific context of cardiac surgery, several RCTs and meta-analyses of RCTs have demonstrated the ability of posterior left pericardiotomy to prevent POAF by limiting the impact of pericardial effusion [38,39]. Indeed, this surgical maneuver drains the pericardial space into the left pleural cavity. Comparing posterior left pericardiotomy to no intervention in 420 patients undergoing conventional cardiac surgery, Gaudino et al. found a significant reduction in POAF in the intervention group (17% vs. 32%; *p* < 0.001) [38]. Similar results were recently reported in a meta-analysis of 25 RCTs [39]. The procedure is now recommended both in American and European guidelines (Class IIa recommendation, level of evidence B) [28,29]. Posterior left pericardiotomy is, however, not yet a routine practice worldwide.

Other medical interventions currently lack scientific evidence or show conflicting results in the scientific literature, so that a wide use for medical practice cannot be recommended. Thus, the prophylactic benefit of magnesium sulfate (while commonly used by care providers), statins, sotalol, steroids, or botulium injection into the epicardial fat pad in cardiac surgery is controversial [32,40,41]. Additionally, the widespread practice that consists of maintaining high–normal serum potassium concentration to avoid POAF after cardiac surgery can be abandoned [42]. Finally, the international multicenter RCT evaluating the effects of colchicine on POAF and myocardial injury in 3209 patients undergoing noncardiac thoracic surgery (COP-AF trial) found no significant reduction in the incidence of clinically important POAF (6.4% vs. 7.5%; HR 0.85 [95% CI: 0.65–1.10]; *p* = 0.22), but did find an increased risk of mostly benign non-infectious diarrhea [43].

## 3. Treatment of POAF

### 3.1. Treatment of Associated Factors

The assessment and management of associated factors contributing to POAF with rapid ventricular response should always be done as a prerequisite in the setting of cardiac and noncardiac surgery (Class IIa recommendation, level of evidence C) [44]. It includes treatment of hypovolemia or fluid overload, electrolytic disturbances such as hypokalemia and hypomagnesemia, hypoxemia, anemia, pain, and management of sepsis or cardiogenic shock. Positive inotropic agents should be avoided when possible.

### 3.2. Considering a Rate Control Strategy

The largest multicenter RCT comparing rate control and rhythm control strategies to treat POAF after cardiac surgery found no significant difference in the total number of hospital days (median, 5.1 days and 5.0 days, respectively; *p* = 0.76), complication rate, and rate of persistent atrial fibrillation 60 days after onset [2]. The conclusion was that neither treatment strategy showed a net clinical advantage over the other. Interestingly, about 25% of patients in the rhythm control group deviated from the assigned therapy because of amiodarone side effects or adverse drug reactions. Since surgical patients with POAF are most often in hemodynamically stable condition (hemodynamics is not compromised by the rhythm disorder), and given that (i) spontaneous return to sinus rhythm is frequent, (ii) amiodarone has serious and complex side effects, (iii) postoperative sympathetic activation is constant, and (iv) beta-blockers are efficient in heart-rate control, a rate control therapy using beta-blockers is recommended as initial strategy in the acute care setting (Class I recommendation, level of evidence B) [29]. Diltiazem or verapamil can also be used as first-choice drugs in patients with left ventricular ejection fraction > 40% to control heart rate and reduce symptoms (Class I recommendation, level of evidence B) [29]. They are preferred over digoxin because of their more rapid onset of action and dose-dependence effects [45,46,47]. Cardio-selective beta-blockers have a better efficacy and safety profile than unselective beta-blockers [48]. Intravenous esmolol and landiolol have the highest cardio-selectivity and shortest elimination half-life, suggesting they may be preferred in acute care patients who are hemodynamically unstable or with severely impaired left ventricular function (Class IIb recommendation, level of evidence B) [29]. Landiolol could both increase and accelerate the rate of conversion of POAF when compared to control following cardiac surgery [35]. In a recent systematic review of new-onset atrial fibrillation in critical care, Levy et al. reported in 324 non-cardiac surgery patients from 17 studies that the use of landiolol was associated with a heart rate decrease of 18% to 51% and a mean conversion time of atrial fibrillation of 1.8 h to 9.1 h with 13% incidence of hypotension [4]. Combination of rate control therapy (cardio-selective beta-blocker or diltiazem/verapamil plus digoxin) should be considered if a single drug does not control symptoms or heart rate. A lenient rate control with a resting heart rate of <110 bpm should be considered as the initial target with stricter control reserved for patients with continuing atrial fibrillation-related symptoms (Grade IIa recommendation, level of evidence B) [29]. Combination of beta-blockers with diltiazem/verapamil should be avoided for safety reasons [49]. Amiodarone may be considered to achieve acute control of heart rate in selected patients who have hemodynamic instability and/or severely reduced left ventricular function or receiving positive inotropic agents (Class IIb recommendation, level of evidence B) [29], but due to its broad extracardiac adverse effects, the drug must be reserved as the last option when heart rate cannot be controlled even with maximal tolerated combination therapy. 

### 3.3. Which Place for a Rhythm Control Strategy in the Acute Care Setting?

**Figure 2:**
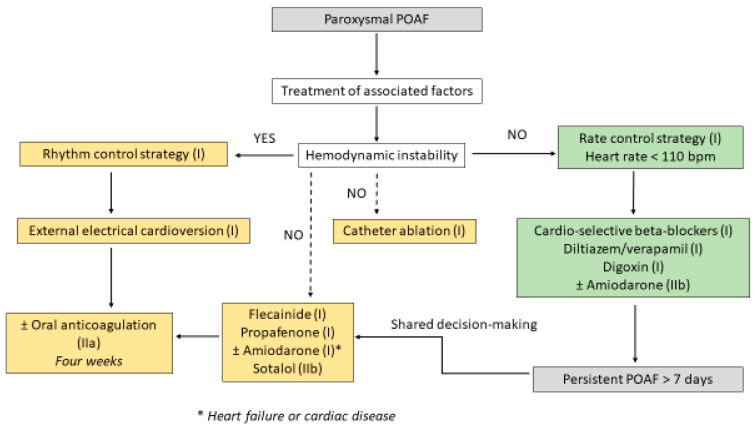
Synthesis algorithm for the treatment of postoperative atrial fibrillation (POAF). In paroxysmal POAF without hemodynamic instability, a lenient rate control strategy (heart rate < 110 bpm) using cardio-selective beta-blockers should be the first-line treatment. Amiodarone may be considered in selected patients who have severely reduced left ventricular function or receiving positive inotropic agents. The decision-making process regarding rhythm control strategy for the treatment of paroxysmal and/or persistent POAF should be shared with cardiologists.

When POAF is responsible for hemodynamic instability (nearly 10% of patients), a rhythm control strategy using rapid external synchronized electrical cardioversion is imperative to improve immediate patient outcomes [4] (Grade I recommendation, level of evidence C) [29]. Additionally, in patients with hemodynamically stable POAF persisting beyond postoperative day 7, a rhythm control strategy is recommended as second-line therapy (Grade IIa recommendation, level of evidence B) [29], and the decision-making process must be shared with cardiologists. Depending on the existence or lack of underlying coronary, valvular, or heart diseases, the antiarrhythmic drug strategy can be amiodarone, dronedarone, flecainide, or propafenone. A synthesis algorithm regarding the treatment of POAF is depicted in Figure 2. Percutaneous catheter ablation procedures may be considered in patients with persistent POAF albeit it is not clear whether first-line ablation is superior to drug therapy.

### 3.4. Prevention of Thromboembolic Events

For a long time, international guidelines have been recommending to initially manage asymptomatic POAF with rate control and anticoagulation. Recently, the 2024 AHA/ACC/ACS/ASNC/HRS/SCA/SCCT/SCMR/SMV guideline for perioperative cardiovascular management for noncardiac surgery suggested that initiation of postoperative anticoagulation therapy can be beneficial in patients with POAF after considering the competing risks associated with thromboembolism and perioperative bleeding (Class IIa recommendation, level of evidence C) [44]. The last version of the CHA_2_DS_2_-VASC risk score can help the decision-making process by calculating the patient’s individual thromboembolic stroke risk [29]. Indeed, the evidence for prevention of POAF-induced ischemic stroke by oral anticoagulants is limited [50,51], whereas the bleeding risk soon after cardiac and major noncardiac surgery is high [51]. Moreover, it is unclear whether stroke mechanisms are the same in patients with POAF compared to those with nonsurgical new-onset atrial fibrillation. A recent meta-analysis reported a significant decrease in thromboembolic events following cardiac surgery when oral anticoagulants were used, at the expense of higher rates of bleeding [52]. Waiting for the results of ongoing RCTs in cardiac and noncardiac surgery, long-term oral anticoagulation should be considered only in patients with POAF at elevated thromboembolic risk to prevent ischemic stroke and thromboembolism (Class IIa recommendation, level of evidence B) [29].

Main studies and meta-analyses on prevention and treatment of POAF following cardiac and noncardiac surgery are reported in Table 1.

## 4. Future Directions

The potential great interest of landiolol as first-line therapy to both prevent and treat POAF after cardiac and noncardiac surgery is the matter of ongoing RCTs whose results should be soon available. The LANDIPROTECT trial is a multicenter, prospective, randomized, controlled, and double-blinded phase III study assessing the protective impact of a 24 h low-dose of landiolol (2 µg/kg/min) administered postoperatively at the arrival in ICU vs. placebo on the incidence of POAF within 7 days following conventional cardiac surgery with cardiopulmonary bypass (NCT04607122). The LANDI–POAF trial is another ongoing multicenter, prospective, randomized, controlled, double-blinded, phase III, and two parallel-arm study assessing the protective impact of a 72 h low-dose of landiolol (2 µg/kg/min) administered postoperatively vs. placebo on the incidence of both POAF and mortality within 72 h following adult cardiac surgery (NCT05084118). The FAAC trial is a multicenter, prospective, randomized, controlled, single-blinded, and two parallel-arm study, which planned to include 350 patients with a first episode of POAF after cardiac surgery. The aim is to compare landiolol to amiodarone with the hypothesis of both a higher rate of reduction to sinus rhythm with landiolol during the 48 h after POAF and less adverse effects within 1 year after surgery (NCT04223739) [53].

The hemodynamic tolerance of POAF is currently defined as the absence of significant hypotension, pulmonary edema, or myocardial ischemia. However, patients experiencing POAF after cardiac surgery without any decrease in mean arterial pressure were found both with a lower pulse pressure (suggesting a decrease in cardiac output) and alterations in microcirculatory parameters during POAF, all values returning to baseline once normal sinus rhythm was restored [54]. Interestingly, intravenous esmolol tended to normalize microcirculatory variables in a dose-dependent manner, and independently of its macrocirculatory effects [54]. Thus, POAF deemed as hemodynamically stable based on arterial blood pressure and usual clinical signs could be responsible for early, occult, and reversible abnormalities in microcirculation [55] that may be improved by short-acting cardio-selective beta-blockers. Further studies are, however, mandatory to figure out the consequences of POAF-induced microcirculatory abnormalities on patients’ outcome and the potential beneficial or deleterious specific pharmacological effects of rate and rhythm control medications, especially beta-blockers.

The direct interrogation of left atrial subtle structural/functional abnormalities by means of preoperative echocardiography has emerged as a new and promising method to identify patients at increased risk of POAF following both cardiac and noncardiac surgery [56,57]. Thus, left atrial strain parameters by preoperative speckle tracking were discriminant in predicting POAF in 310 patients undergoing coronary artery bypass grafting (area under the ROC curve 0.74; *p* < 0.001) [56]. Additionally, left atrial strain metrics might aid with identifying patients undergoing lung resection who are high-risk and benefit from prophylactic therapy [57].

SGLT2 inhibitors are increasingly used in patients with diabetes and chronic heart failure. A recent meta-analysis of 52 RCTs including 112,031 patients showed a protective effect against atrial fibrillation depending on the baseline clinical condition [58]. Further studies conducted in cardiac and noncardiac surgical patients are mandatory to assess their potential role in preventive POAF.

Finally, the definite benefit/risk ratio and optimal long-term use of oral anticoagulation among patients with POAF should be better informed by the results of ongoing RCTs conducted in cardiac surgery (NCT04045665) and noncardiac surgery (NCT03968393).

## 5. Conclusions

POAF is common after cardiac and major noncardiac surgery and significantly associated with short- and long-term adverse events, thereby justifying an efficient proactive strategy of both prevention and treatment. Despite its great popularity among care providers worldwide, discrepancies regarding the indications of amiodarone exist in the most recent international guidelines. Considering its serious side effects and complex drug interactions, amiodarone should be reserved for patients at high risk of POAF or with hemodynamic instability or severely reduced left ventricular ejection fraction. Perioperative optimization of cardio-selective beta-blockers to prevent POAF and control heart rate when POAF occurs is the recommended first-line strategy, in association with the treatment of associated factors. Ultra-short-acting intravenous beta-blockers like esmolol or landiolol could be preferentially used in acute care patients. Besides waiting for the results of ongoing RCTs in cardiac and noncardiac surgery, the use of oral anticoagulation in patients with POAF should take into account the individualized thromboembolic/hemorrhagic risk ratio.

## Figures and Tables

**Table 1:** Main studies and meta-analyses on prevention and treatment of postoperative atrial fibrillation (POAF) following cardiac and noncardiac surgery.

First Author	Journal	Study Design
Gillinov AM [2]	N Engl J Med 2016	Multicenter RCT comparing rate control vs. rhythm control for POAF after cardiac surgery
Mathew JP [5]	JAMA 2004	Large cohort study showing a significant association between perioperative optimization of beta-blockers and POAF
Isiadinso I [30]	J Heart Lung Transplant 2011	Retrospective study showing the association between amiodarone and mortality in POAF after heart–lung transplantation
Couffignal C [33]	Anesthesiology 2020	Prospective multicenter cohort study assessing timing of beta-blockers reintroduction and POAF after cardiac surgery
Blessberger H [34]	Cochrane Database Syst rev 2019	Meta-analysis reporting the role of beta-blockers for preventing morbidity and mortality after noncardiac surgery
Cafaro T [36]	Can J Anesth 2023	Meta-analysis of RCTs showing the efficacy of landiolol in preventing POAF after cardiac surgery
Kaminohara J [37]	JTCVS Open 2022	Propensity score-matched analysis showing the efficacy of landiolol in preventing POAF after cardiac surgery
Gaudino M [38]	Lancet 2021	RCT showing the efficacy of posterior left pericardiotomy for the prevention of POAF after cardiac surgery
Curran J [41]	Plos One 2023	Meta-analysis showing the conflicting results regarding magnesium prophylaxis of new-onset atrial fibrillation
O’Brien B [42]	JAMA 2024	Multicenter RCT showing the lack of effect of potassium supplementation to prevent POAF after cardiac surgery
Conen D [43]	Lancet 2023	International RCT showing the lack of effect of colchicine to prevent POAF after noncardiac surgery
Taha A [51]	JAHA 2021	Population-based nationwide registry studying POAF and long-term outcome after cardiac surgery
Neves IA [52]	Vascul Pharmacol 2022	Meta-analysis on the use of anticoagulation in POAF after cardiac surgery

RCT: randomized controlled trial.
